# Supplementary material for: Early detection of new pandemic waves. Control chart and a new surveillance index
Source: PLoS One. 2024 Feb 12;19(2):e0295242. doi: 10.1371/journal.pone.0295242 (PMC10861055; doi:10.1371/journal.pone.0295242)
Supplement: S1 Appendix — (PDF) [file pone.0295242.s001.pdf]

APPENDIX A. Steps in the construction of the deseasonalized data series X(t) and calculation of the seasonal factors f_L, f_H y f_M

We have implemented the following deseasonalisation method included in [46].

1. The series $\bar{Y}_7(t)$ of moving averages of the original data series $Y(t),\ t = 1, \dots, n$ is calculated using seven terms, assuming a one-week period of oscillation.

$$\bar{Y}_7(t) = \sum_{j=-3}^{+3} Y(t+j)\ /7 \quad \forall t = 4, \dots, n-3$$

2. The original data $Y(t)$ are divided by the results from step 1, to obtain the daily factors, distinguishing among three types of days: non-Monday, non-post-holiday working days (set L), holiday and weekend days (set H) and Mondays and post-holiday days (set M).

$$f_L(t) = Y(t)/\bar{Y}_7(t) \ \ \forall\, t \in L$$

$$f_H(t) = Y(t)/\bar{Y}_7(t) \ \ \forall\, t \in H$$

$$f_M(t) = Y(t)/\bar{Y}_7(t) \ \ \forall\, t \in M$$

3. The average of seasonal factors is computed to obtain the seasonal components

$$f_L = \sum_{t \in L} f_L(t)/\#L$$

$$f_H = \sum_{t \in H} f_H(t)/\#H$$

$$f_M = \sum_{t \in M} f_M(t)/\#M$$

4. The original data series $Y(t)$ is divided by the (adjusted) seasonal factors to obtain the series $X(t)$ of **deseasonalized data**.

$$X(t) = Y(t)/f_D \qquad D = L\,, H, M \ \ for\ t \in L, H, M, respectively$$

[46] Box GEP, Jenkins GM, Reinsel GC, Ljung GM. *Time Series Analysis: Forecasting and Control*. Wiley, 2015.
