# Supplementary material for: Early detection of new pandemic waves. Control chart and a new surveillance index
Source: PLoS One. 2024 Feb 12;19(2):e0295242. doi: 10.1371/journal.pone.0295242 (PMC10861055; doi:10.1371/journal.pone.0295242)
Supplement: S2 Appendix — (PDF) [file pone.0295242.s002.pdf]

APPENDIX B. Mathematical proofs for the Household Cluster Probabilistic (HCP) model.

Proposition 1. The mean and variance of the variable number of new cases $Y(t) = \sum_{i=1}^{F(t)} C_i$, in the HCP model where C_i are $E(C) = \mu_C = \mu_{F(t)}\mu_C$ and variance $V(C) = \sigma_C^2 = \mu_{F(t)}E(C^2)$.

Proof. Assuming that $F(t)$ has a Poisson distribution, then Wald's equation provides the expected value of $Y(t)$:

$$E(Y(t)) = E\left(\sum_{i=1}^{F(t)} C_i\right) = E\big(F(t)\big)E(C) = \mu_{F(t)}\mu_C$$

To obtain the variance expression, we use the law of total variance,

$$\begin{aligned} V(Y(t)) &= E\Big(V\big(Y(t)\big|F(t)\big)\Big) + V\Big(E\big(Y(t)\big|F(t)\big)\Big) = E\big(F(t)V(C)\big) + V\big(F(t)E(C)\big) \\ &= V(C)E\big(F(t)\big) + E(C)^2V\big(F(t)\big) = \sigma_C^2\mu_{F(t)} + {\mu_C}^2\mu_{F(t)} = \mu_{F(t)}(\sigma_C^2 + {\mu_C}^2) \\ &= \mu_{F(t)}E(C^2) \end{aligned}$$

Corollary 1. The HCP model is overdispersed: $V(Y(t)) > E(Y(t))$.

Proof. The random variable, C, for the number of infected members of an infected household is, by definition, C≥1, because a household is considered infected when at least one of its members is infected Then, because the variable can take a value greater than one with a positive probability ($P\{C > 1\} > 0$), then $E(C^2) > E(C)$ and therefore, from the previous proposition the results follows: $V(Y(t)) > E(Y(t))$.

Proposition 2. The probability that a household is infected on a particular day is proportional to the number of its inhabitants.

Proof. It is assumed that an individual becomes infected on day t with probability p, which is very small ($p \ll 1$) and equal for all individuals of the population.

$$P\{household\ of\ size\ k\ is\ infected\} = 1 - (1-p)^k \approx kp\ (using\ Taylor\ expansion)$$

The following result shows that the probability distribution of infected households I_H only depends uniquely on the household size distribution H.

Proposition 3. The probability distribution of variable I_H, size of infected households, is

$$P\{I_H = k\} = \frac{kP\{H = k\}}{\mu_H}$$

where H denotes the household size variable.

Proof. Assuming that all individuals in the population have the same probability p of being infected and applying the Laplace rule:

$$P\{I_H = k\} = \frac{kP\{H = k\}}{\sum_{i=1}^{\infty} iP\{H = i\}} = \frac{kP\{H = k\}}{\mu_H}$$

The following result provides expressions for $\mu_{F(t)}$ and $E(C^2)$ for the evaluation of the variance:

$$V(Z_P(t)) = \frac{\alpha^2}{(1-(1-\alpha)^P)^2} \sum_{j=0}^{P-1} (1-\alpha)^{2j}\, \mu_{F(t)} E(C^2)\big(f_L 1_{\{t\in L\}} + f_H 1_{\{t\in H\}} + f_M 1_{\{t\in M\}}\big)$$

Proposition 4. Assuming that g, the ratio of new home infections on total population, and H, the household size distribution, are known, then

$$\mu_{F(t)} = (1-g)\, E(Y(t))$$

$$E(C^2) = \frac{\mu_H E(H^3)}{E^2(H^2)(1-g)^2}$$

Proof. By the definition of the ratio , $\mu_{F(t)} = (1-g)\, E(Y(t)$.

From Proposition 1, $E(Y(t)) = \mu_{F(t)}\mu_C$. Then, $\mu_{F(t)}(\mu_C - 1) = g\, E(Y(t))$ and $\mu_C = \frac{1}{1-g}$.

Using the probability distribution of variable H, it is possible to calculate the mean and second moment of the variable I_H, size of infected households:

$$\mu_{I_H} = \frac{\sum_k k^2 P(H = k)}{\sum_k kP(H = k)} = \frac{E(H^2)}{\mu_H}$$

$$E({I_H}^2) = \frac{\sum_k k^3 P(H = k)}{\sum_k kP(H = k)} = \frac{E(H^3)}{\mu_H}$$

The variable C, number of infected members in each infected household, is related with the size of the infected household: $C = \gamma(I_H)$. Assuming that all members of a household become infected, then $C = I_H$, and

$$\mu_C = \mu_{I_H} = \frac{E(H^2)}{\mu_H}$$

$$E(C^2) = E({I_H}^2) = \frac{E(H^3)}{\mu_H}$$

It can also be assumed that not all members of an infected household will become infected. Linearly approximating this relationship: $C = hI_H$, then

$$\mu_C = h\frac{E(H^2)}{\mu_H}$$

$$E(C^2) = h^2\frac{E(H^3)}{\mu_H}$$

Taking into account that $\mu_C = h\frac{E(H^2)}{\mu_H} = \frac{1}{1-g}$, then $h = \frac{\mu_H}{(1-g)E(H^2)}$, and $E(C^2) = \frac{\mu_H E(H^3)}{E^2(H^2)(1-g)^2}$
