# Supplementary material for: Early detection of new pandemic waves. Control chart and a new surveillance index
Source: PLoS One. 2024 Feb 12;19(2):e0295242. doi: 10.1371/journal.pone.0295242 (PMC10861055; doi:10.1371/journal.pone.0295242)
Supplement: S3 Appendix — (PDF) [file pone.0295242.s003.pdf]

APPENDIX C. R-code

```
# This function generates the data-frame that contains the data to build the EWMA Control Chart.
# INPUTS:
# MdiaControl <-  is a data frame with at least the variables:
#            DIA: date in format aaaa-mm-dd.
#            SerieDes3: Desasonalised value of the data.
#            tipoDia3f: type of days (labourable, non-labourable, etc.)
# dinicio <- Start point of the training period (aaaa-mm-dd).
# dfinal <- End point of the training period (aaaa-mm-dd).
# lambda <- value of the lambda parameter.
# factores3 <- table with the deseasonalized factor by type of day.
# PesoCluster <- value of the parameter of weith of the clusters.
# g <- probability of infections within home.

# OUTPUT:
# MdiaEst <- data frame with the next variables:
#         DIA: date in format aaaa-mm-dd.
#         Zi: value of the WAI index.
#         Vari: value of the variance.
#         LCS: upper limit of the Control Chart.
#         LCI: lower limit of the Control Chart.


EWMAControlChart <- function (MdiaControl,dinicio, dfinal,lambda=0.2,L=3,factores3=factores3,g=0.4){


  # Moments of order 1, 2, 3 of the variable with the distribution of home size in Spain
  Datos <- data.frame(Size=c(1:8),Frec=c(4889900,5703700,3845800,3219800,685300,252000,100400,58000))
  #data, frequency and probability
  Datos <- Datos %>% mutate(Prob=Frec/sum(Frec))
  sum(Datos$Prob)
  #E(H)
  muH <- sum(Datos$Size*Datos$Prob)
  #E(H^2)
  muH2 <- sum(Datos$Size^2*Datos$Prob)
  #E(H^3)
  muH3 <- sum(Datos$Size^3*Datos$Prob)
  ### Use g as probability of whitin home infectious.
  muC <- 1/(1-g)
  # muC=h*E(C^2)/muH
  h <- muC*muH/muH2
  #calculus of E(C^2)
  EC2 <- h^2*muH3/muH

  MdiaEst<- MdiaControl
  MdiaEstLimCalc <- MdiaControl %>% filter(DIA<=dfinal)

  #Zi calculus
  MdiaEst$Zi <- 0
  MdiaEstLimCalc$Zi <- 0
  MatrizCoeficientes <- matrix(NA,nrow=nrow(MdiaEst),ncol=(nrow(MdiaEst)+1))
  for(i in 1:nrow(MdiaEst)){
```

```
    sumi <- 0
    coefeces <- NULL

    P_1 <- min(i-1,13)
    for(j in 0:P_1){
      sumi <- sumi+(1-lambda)^(j)*MdiaEst$SerieDes3[i-j]
      coefeces <- c(coefeces,(1-lambda)^(j))
    }
    MdiaEst$Zi[i] <- (lambda/(1-(1-lambda)^(P_1+1)))*sumi
    if(i<=nrow(MdiaEstLimCalc)){
      MdiaEstLimCalc$Zi[i] <-MdiaEst$Zi[i]
    }
    coefeces <- c((lambda/(1-(1-lambda)^(P_1+1)))*coefeces)
    MatrizCoeficientes[i,1:(P_1+1)] <- coefeces

  }

  N <- mean(MdiaEstLimCalc$Zi)
  muF <- mean(MdiaEstLimCalc$SerieDes3)

  #write.table(MatrizCoeficientes,file="MatrizDeCoeficientes.txt",sep="\t",col.names =
FALSE,row.names = FALSE)

  #Variance calculus
  MdiaEst$Vari <- 0
  MdiaEstLimCalc$Vari <- 0

  for(i in 1:nrow(MdiaEst)){
    P_1 <- min(i-1,13)
    sumi <- 0
    for(j in 0:(i-1)){

      sumi <- sumi+(1-
lambda)^(2*j)*factores3$FactPond[factores3$tipoDia3f==MdiaEst$tipoDia3f[i-j]]*(1-
g)*muF*EC2  #*N*PesoCluster
    }
    MdiaEst$Vari[i] <- (lambda^(2)/(1-(1-lambda)^(P_1+1))^(2))*sumi#
    if(i<=nrow(MdiaEstLimCalc)){
      MdiaEstLimCalc$Vari[i] <-MdiaEst$Vari[i]
    }
  }

  if(nrow(MdiaEst)>=(nrow(MdiaEstLimCalc)+1)){
   for(i in (nrow(MdiaEstLimCalc)+1):nrow(MdiaEst)){
    MdiaEst$Vari[i]<-MdiaEst$Vari[i-7]
  }
  }

  MdiaEst$LCS <- N+L*sqrt(MdiaEst$Vari)
  MdiaEst$LCI <- N-L*sqrt(MdiaEst$Vari)
  MdiaEst <- MdiaEst %>% mutate(LCI=ifelse(LCI<0,0,LCI))


  return(MdiaEst)
}
```
